# Implementing exergames into healthcare for chronic conditions – insights from stakeholders: a qualitative study

**DOI:** 10.1186/s12961-025-01416-7

**Published:** 2025-10-30

**Authors:** Marianna Antoniadou, Aurel Zelko, Anna Strömberg, Tiny Jaarsma, Leonie Klompstra

**Affiliations:** 1 Department of Health, Medicine and Caring Sciences, Linköping University, 581 83 Linköping, Sweden; 2 Department of Health Psychology and Research Methodology, Faculty of Medicine, Pavol Jozef Safarik University, 040 11 Kosice, Slovakia; 3 Department of Cardiology, Linköping University, 581 83 Linköping, Sweden

**Keywords:** SWOT analysis, Exergaming, Implementation, Stakeholders, healthcare

## Abstract

**Background:**

Exergaming, which combines physical exercise with video gaming, has shown benefits for individuals with chronic conditions. Implementation occurs at different levels and phases and influenced by various factors. To address the factors that influence the implementation of exergaming in healthcare, we aimed to explore stakeholder’s experiences of strengths, weaknesses, opportunities and threats to the implementation of exergaming (in preparation, execution and continuation) for individuals with chronic conditions, in healthcare at the micro, meso and macro level.

**Methods:**

A qualitative study with deductive content analysis was performed to explore stakeholder’s experiences regarding the strengths, weaknesses, opportunities and threats (SWOTs) of implementing exergaming in healthcare. Data were collected through semi-structured interviews with 24 stakeholders, including patient representatives, researchers, healthcare professionals, game developers and individuals involved in healthcare regulations.

**Results:**

At the micro level assessing patient’s needs and involving stakeholders in the development and evaluation phases were described as strengths in implementing exergaming in healthcare. Weaknesses included patient’s lack of digital literacy and healthcare professional’s concerns about the safety and quality of exergames. The involvement of healthcare professionals and family support were described as opportunities, whereas threats included the challenge of tailoring exergames to patient’s needs and healthcare professional’s fear of losing control over the technology. At the meso level, strengths involved collaboration between healthcare professionals and technicians, whereas weaknesses included the high cost and time required for designing exergames. Opportunities were found the use of existing exergames, and threats involved competition for research grants and staff turnover. At the macro level, strengths included supportive regulations and collaboration among policy-makers, whereas weaknesses involved defining intended use and fragmented responsibilities. Opportunities were identified in governmental funding programs and international collaborations, whereas threats included challenges in data storage and sharing.

**Conclusions:**

The effective implementation of exergaming in healthcare requires coordinated efforts. Stakeholder’s involvement, supportive leadership and digital readiness are crucial for successful implementation, while inconsistent implementation of the policies and limited evidence on patient safety pose significant barriers. These insights can inform future strategies for integrating exergaming into healthcare settings.

**Supplementary Information:**

The online version contains supplementary material available at 10.1186/s12961-025-01416-7.

## Introduction

Exergaming has gained popularity as a relatively new tool to increase motivation for regular physical activity among sedentary individuals, including individuals with chronic conditions such as type 2 diabetes mellitus and heart failure [1, 2]. Exergaming is defined as playing a videogame by using the full body, and it requires the end user to expend a significantly greater amount of energy than resting levels do [3]. A growing body of research highlights the positive impact of exergaming on physical health in individuals with chronic conditions, including improvements in physical capacity [4], balance [5], muscle strength [6] and mobility [5].

Evidence for exergaming in chronic disease management remain mixed. In cardiac rehabilitation, Blasco-Peris et al. (2022) reported that exergame-based programs offered no significant advantage over conventional approaches in exercise capacity, quality of life or mental health, with inconsistent effects on long-term adherence [7]. In contrast, findings from neurological rehabilitation appear more promising: Moeinzadeh et al. (2023) reported that virtual reality (VR) exergaming in patients with multiple sclerosis often led to greater gains in physical and cognitive abilities, psychosocial outcomes and fatigue [8], while Marotta et al. (2022) observed improvements in executive function and other cognitive domains in patients with Parkinson’s disease [9]. However, these results were tempered by small sample sizes and methodological limitations.

The effective implementation of exergaming in healthcare for individuals with chronic conditions depends on factors such as organizational and system readiness as well as user acceptance of the technology [10]. In general, digital solutions are more likely to be adopted by healthcare professionals and patients when they demonstrate high usability and can be tailored to both the target population and individual preferences and needs [11]. Usability is the ability of a system or device to be used easily and effectively [12].

To support the implementation of good practices across different countries, the European Joint Action on Chronic Diseases and Promoting Healthy Ageing across the Life Cycle (JA-CHRODIS) was launched in 2014 [13], followed by CHRODIS+ in 2017 [14]. Their aim is to support international and European goals by promoting the exchange and implementation of good practices across countries to improve chronic condition management [15]. Despite the presence of policies and regulations that can support the integration of innovations in healthcare, including exergaming, their practical implementation remains challenging [16]. One example of these regulatory requirements is the European Union’s Medical Device Regulation (MDR), under which whether an exergame, or another device, qualifies as a medical device depends on its intended use [17]. This means that, if the exergame is intended to be used as a medical device, it must obtain Conformité Européenne (CE) marking under the MDR [18]. However, if the developer does not clearly define the exergame’s intended medical use, it creates regulatory uncertainty and potential delays in implementation.

An underlying barrier to implementation of exergaming can be the lack of stakeholder’s involvement or other possible parties (such as individuals involved in regulations within healthcare) in the development of exergames [19]. The possible stakeholders involved in the development and implementation of exergames are end users, healthcare professionals, researchers and game designers/developers [19]. Other reasons for unsuccessful implementation can include the negative attitudes of patients and healthcare professionals toward technology, a lack of focus on patient’s needs, a lack of adequate cost–effectiveness studies and a lack of evidence about existing exergames [20, 21].

Exploring the strengths, weakness, opportunities and threats (SWOTs) of implementing exergames in healthcare has previously been used to assess interventions within healthcare and to facilitate the performance and quality of service delivery [22–24]. A SWOT framework can be used to address the internal [strengths (S) and weaknesses(W)] and external [opportunities (O) and threats (T)] factors that either facilitate or constrain implementation.

The implementation process can be viewed across several distinct levels: the micro level, which focuses on direct interactions between patients and healthcare professionals; the meso level, which pertains to organizational and institutional frameworks; and the macro level, which includes the broader healthcare system, involving laws and regulations [16]. Influencing factors vary at each level, for instance, individual characteristics at the micro level, organizational structures at the meso level and policy and legal frameworks at the macro level.

In addition, implementation also occurs in phases that should be considered when developing and implementing exergames in healthcare. The theory of adaptive implementation of care innovations, in combination with levels, outlines three main implementation phases: the phase of preparation (during game development or initiation), execution (actual implementation) and continuation (stabilization) [25]. This theory was previously used to identify barriers to and facilitators of implementing exergaming for people with dementia in day care centres [20]. It can also be meaningful to use this theory to further explore the SWOT of implementing exergaming in a broader context for individuals with various chronic conditions in different levels of healthcare.

A facilitator in one phase or level can act as a barrier in another phase [26]. Despite the growing body of research on exergaming, there remains a gap in understanding how implementation unfolds across various levels and phases. The literature tends to emphasize outcomes on stakeholder’s experiences in isolated contexts, often overlooking the complex, multiphase nature of deploying exergaming in healthcare [27–29]. The exploration of these factors can assist game developers in developing successful exergames, healthcare professionals in using exergames properly in practice, individuals involved in regulations within healthcare in establishing guidelines that support the use of exergaming in healthcare and researchers in collaborative and inclusive research strategies.

### Aim

In this study, we aim to explore stakeholder’s experiences of strengths, weaknesses, opportunities and threats in the implementation of exergaming (in preparation, execution and continuation) for individuals with chronic conditions, in healthcare at the micro, meso and macro levels.

## Methods

### Study design

This is a qualitative study using deductive content analysis. Data collection and analysis were inspired by the theoretical model of adaptive implementation to trace strengths, weaknesses, opportunities and threats in the preparation, execution and continuation phases [25]. The Consolidated Criteria for Reporting Qualitative Research (COREQ) checklist was used to ensure complete and transparent reporting of this qualitative study [30].

### Sampling strategy and participants

A purposeful sampling strategy including snowball sampling was used to recruit stakeholders who varied in terms of their country of origin, age, years of experience with exergaming and level of familiarization with these tools. In this study, stakeholders were considered to be end users (patient representatives), researchers, healthcare professionals, game developers and individuals involved in healthcare regulations.

The participants had to meet at least one of the following criteria: being involved in the development or design of exergames and/or other digital technologies for individuals with chronic conditions, researching exergaming for individuals with chronic conditions, using exergaming and/or other digital technologies when working with individuals with chronic conditions in their daily practice, developing regulations and policies regarding exergaming in healthcare or serving as a patient representative (chronic conditions represented were heart and/or lung disease, rheumatism, nephrology and haemodialysis). Some of the researchers included in this study held different roles, combining clinical work with research in digital technologies and/or exergaming for this population. Study participants who could speak English, Swedish or Slovakian were eligible for the study, as these languages were accessible within the research team. The researchers screened the potential participants to verify whether they fulfilled the inclusion criteria before inviting them to participate in the study.

Researchers emailed potential study participants with study details and interview information, and an online interview was scheduled after the participants expressed interest. A total of 24 participants were interviewed, and information power was achieved after the 20th participant because of clear redundancy with repetition of patterns in the data without novel additions [31]. No participants declined participation or discontinued their involvement in the study. A table presenting the demographic characteristics of the participants is provided in Table 1.

**Table 1:** Participant characteristics (*n* = 24)

Demographics	
Age (median, IQR)	53 (43–77)
Female sex (*n*)	11
Male sex (*n*)	13
Participant’s role^*^	
Patient representative (*n*)	5
Researcher (*n*)	10
Healthcare professional (*n*)	7
Game developer (*n*)	5
Involved in regulations within healthcare (*n*)	9
Country (*n*)	
Sweden	10
Slovakia	2
USA	3
Australia	2
Iceland	2
Israel	1
Belgium	2
The Netherlands	2
Years of experience with exergaming (median, IQR)	9.5 (5–25)

IQR, interquartile range

^*^More than one answer possible

### Data collection

The interview guide was drafted by three of the authors (M.A., A.Z. and L.K.) and then reviewed in discussion with the full author team. Its questions were informed by the literature [20], they were adapted on the basis of the SWOT framework, and specific questions were asked about whether strengths, weaknesses, opportunities and threats were experienced at the micro, meso or macro level. To ensure that all participants understood the phenomenon of exergaming, before the start of the interviews, the following definition of exergaming was provided and discussed: Exergaming can be defined as playing a videogame by using full body movement to control on-screen action, and it requires the player to expend a significantly greater amount of energy compared with resting levels. Thus, this type of gaming combines physical exercise and the use of virtual reality and technology. Video game consoles, virtual reality systems or mobile applications are used to support physical activity and provide a more interactive experience (for example, you might have heard of Nintendo Wii or cybercycling). Probing questions such as “Could you elaborate?”, “Could you provide an example?” or “What do you mean by saying that?” were used to facilitate the conversation and provide more accurate descriptions and rich information. The interview guide and the definition of exergaming are provided in Appendix A.

A pilot interview was first conducted with a potential study participant to assess the clarity of the questions. Since no further modifications were needed in the interview guide and the information gathered was highly relevant, the pilot interview was included in the final analysis. All the interviews were recorded through Zoom and transcribed verbatim. One participant requested to read the transcription before the use of the interview in the analysis. This participant agreed to the use of the interview without changes in the manuscript but requested to keep the specific names of the technical solution out of the results, as this would break the anonymity of the participants.

The data were collected through semi-structured interviews. The mean duration of the interviews was 44.8 min. Most of the interviews were conducted in English (*n* = 20) by the two first authors, who contributed equally to the data collection. Although both interviewers had previous experience with interview studies, their experience was limited. To strengthen the process, a pilot interview was conducted, and the resulting transcripts were shared with the research group for feedback. Two interviews were conducted in Swedish by a researcher who is an experienced interviewer, and two interviews were conducted in the Slovakian language by the second author. The interviewers had no previous knowledge or relationships with the study participants. Twenty participants were interviewed one by one, whereas two joint interviews were conducted, due to participant’s requests, since they belonged to the same research group. No field notes were kept by the researchers during the process of data collection.

### Data analysis

The data were analysed via deductive content analysis, following the guidelines outlined by Elo and Kyngäs (2008) [32]. This form of analysis is used when the structure of analysis is based on a pre-existing framework or theory. In this study, the deductive analysis was based on a SWOT analysis and was inspired by the model of adaptive implementation [25].

First, three of the authors (M.A., A.Z. and L.K.) read through the transcribed interviews to familiarize themselves with the data and gain an in-depth understanding of the data. The first three interviews were subsequently analysed in parallel by these authors. The remaining interviews were independently analysed by the first author.

The participant’s responses regarding the use of exergaming were structured into strengths, weaknesses, opportunities and threats. SWOT analysis was performed at the micro, meso and macro levels (on the basis of the model of adaptive implementation [25]) [22]. After identifying the strengths, weaknesses, opportunities and threats at the micro, meso and macro level, we assigned whether these occurred in the preparation, execution or continuation phase of implementation (on the basis of the model of adaptive implementation). NVivo15 software was used to facilitate the coding process. Finally, the findings were presented in alignment with the structured categories, in a way that ensured clarity.

Methodological rigour was ensured by following the guidelines established for qualitative studies to ensure trustworthiness by Lincoln and Guba (1989) [33]. Analysis triangulation was ensured through regular discussions between the authors with the aim of reaching a consensus while considering the categorization of the data. The interviews were read multiple times to obtain an overall understanding of the content, and the parts of the interviews were in constant comparison with the whole. Authentic quotations were used and presented as part of the results to increase the trustworthiness of the findings.

## Results

### SWOT at the micro level for the implementation of exergaming in healthcare

The factors at the micro level are those that are related to the stakeholders, such as their involvement in the different phases of implementation.

*Strengths*: In the preparation phase, participants referred to the significance of assessing patient’s needs when developing a new technology. The involvement of different stakeholders was considered critical both in the preparation and execution phases, when developing, testing and evaluating exergaming. The participants mentioned that the input of patients and their caregivers should be incorporated into the process, which might lead to more effective solutions. The participants described a variety of ways of involving patients and their caregivers in the process, such as patient’s advisory board exergaming, cocreation sessions with patients, discussions at conferences and the involvement of patient’s organizations and associations. The importance of involving different stakeholders during the preparation and execution phases was mentioned:

I think you need to keep stakeholders involved not only in the development phase, but also in the improvement phase. Like when you have the prototypes and the first versions you might come from the side with new ideas, but when you reach a more iterative making continue small improvement. I think many of those improvements should come from feedback from the patients, and other stakeholders who use the new technology. [Participant (P) 12 – a physician–researcher].

In the execution and continuation phases the participants referred to the importance of introducing simple and interesting exergames. The participants also highlighted the importance of providing options for different games to patients to make them more fun and motivating. The use of simple language when explaining exergames or conducting follow-ups was considered necessary. When exergaming was adapted to patient’s daily activities, the possibility of using it in the continuation phase significantly increased.

When you know exactly how the app, or the game works, then I think it’s nice and fun to play. Then you have something to do. So, in our age you have to make something of it because otherwise, yeah. And the days go and go and there is no meaning. You then you have something to do, you can read, but you can also use the app then. (P20 – a patient representative).

*Weaknesses:* The digital literacy of patients was often characterized as insufficient and participants mentioned that patients are usually more familiar with visiting the healthcare setting, rather than using new technologies. This factor, in combination with healthcare professional’s lack of confidence in the use of exergaming, was considered a weakness at the micro level. Healthcare professionals also expressed their concerns regarding the safety of the patient and the quality of the healthcare service provided. A participant referred to an incident when she was under psychological stress when her patients were exergaming at home:

For about 6 months I didn’t sleep at night because I was afraid that patients would fall when they were playing, but nobody fell and I felt it was really important for them because if they stand, it’s more naturalistic and they can improve their balance as well. If they’re sitting, it’s very restrictive. (P11 – an occupational therapist and researcher).

*Opportunities:* In the execution and continuation phase, an opportunity mentioned was the involvement of healthcare professionals since patients tend to trust them when they suggest exergaming. Having healthcare professionals on board was considered highly important; specifically, a participant mentioned the following:

But I think in general the public they trust healthcare providers a lot. You know they trust them for their lives. So, if we say that we trust the game and we believe it can be helpful, then I think people are more willing than if you just see an advertisement. What is meant in the newspaper? So that’s why you have to have the staff on board. (P2 – a nurse and researcher).

Family member’s support was also considered important since they can understand patient’s needs and can assist patients in using exergames in the long run. Another opportunity was the presence of a large variety of exergames on the market that could be tailored to patient’s needs and used in healthcare. Thus, participants mentioned that perhaps no more exergames need to be developed, but existing games could be adjusted and used in patients with different conditions.

*Threats:* A threat mentioned is that the existing exergames are seldom tailored to patient’s needs and making adjustments could be challenging and time-consuming. Patients diagnosed with more than one chronic condition usually experience severe symptoms which might restrict the use of exergames, according to participants. The participants referred to patient’s involvement as challenging, and approaching patients with diverse needs was characterized as difficult. Moreover, healthcare professionals expressed their fear of losing control over exergaming. An example is provided:

Because they didn’t want to use it because they felt that they were at risk of losing face in front of the patient. And it’s a very sensitive thing for clinicians to kind of lose the respect or whatever you would call it from the patients. So then when once you’ve lost it, it’s going to be very tricky for you to get it back. (P5 – a healthcare professional and researcher).

### SWOT at the meso level for the implementation of exergaming in healthcare

The meso level is related to the organizational and structural processes that affect the implementation of exergaming, involving the collaboration between different professionals and organizations, the needs within the healthcare system and finances.

*Strengths*: During the preparation phase, a strength mentioned at the meso level was the careful assessment of the needs of healthcare professionals. An assessment of the need within healthcare might lead to the development of effective exergames that are more likely to be used by healthcare professionals in the future. The implementation of exergaming should be led by the profession with respect to “the environment and culture of healthcare” mentioned by P17, a person involved in developing regulations within healthcare. Another person involved in developing regulations described how they used role-playing to illustrate how exergaming will be implemented in the future in a specific healthcare setting to facilitate readiness to change:

We create a scenario and then we can theoretically or in role play or in a cardboard work with this scenario, how will this be executed by us? So, you have to play the role in the scenario so that you make it more believable. The healthcare staff has the scenarios they have to write it down so that you can make the readiness or the setting as good as possible. (P15 – a person involved in developing regulations within healthcare).

Establishing and sustaining collaboration between healthcare professionals and technicians during all phases of implementation was also considered a strength. P2, a nurse and researcher, mentioned that technician’s knowledge of “how to make an exergame beautiful” can be complementary to healthcare professional’s understanding of patient’s needs and the requirements of a healthcare setting. Furthermore, developing a time plan and predicting the costs were considered strengths for developing and implementing exergaming in healthcare.

*Weaknesses*: In the preparation phase, a weakness emphasized by participants was that designing an exergame can be expensive and time consuming. The main reason for this is that the need to develop exergames should be appealing but also user-friendly. Ensuring funding to sustain the use of exergaming was also considered challenging. While it might be easier to receive funding to develop an exergame, participants emphasized the challenges of obtaining funding in the execution and/or the continuation phase. A participant also mentioned that making a profit from using exergaming might take time:

We have to work a lot to actually be able to even get money from doing this. And meanwhile, we would be the only ones spending money on it and not getting any money in return. (P7 – a game developer).

*Opportunities*: The participants referred to the opportunity to use existing exergames instead of investing time and money in developing and designing new appealing exergames. A healthcare professional who is also a researcher described the process of tailoring existing games to patient’s needs, using existing evidence. In the execution and continuation phases, participants highlighted that in several healthcare settings there is a welcoming structure in regard to the use of new technologies in clinical practice which could be considered an opportunity for easier implementation when implementing exergaming. In some healthcare settings there is a strong academic culture and a better access to technology.

We did see a difference in their access to technology. If at their workplace they had technology and the Internet was good and they could use it, then there was a bigger chance that they would use. It and at places where it was difficult, they didn’t have that, or the Internet was difficult then they didn’t use it. (P11 – an occupational therapist and researcher).

*Threats:* A threat identified at the meso level was the difficulty of convincing some healthcare settings to use exergaming in everyday practice. Staff rotation or turnover, a lack of resources, the absence of leadership support and an overburdened health workforce were some of the challenges mentioned by the participants. A participant stressed the challenges of leadership:

So we need to have this combined with the implementation in the healthcare system as much as we can and that’s the big problem. For us often the issue is the staff, we need to implement a digital tool is not a technology issue actually because it’s an organizational issue and it’s about leadership and all those things because it’s a new way of working. (P17 – a person involved in developing regulations within healthcare).

Receiving research grants was considered competitive in the preparation phase and existing exergames created even more competition when developing a new one. A lack of evidence regarding the effectiveness of existing exergames was considered a threat, which increased scepticism among healthcare professionals about their use. Finally, the participants thought that the involvement of many people when implementing exergaming in the healthcare setting was a threat.

### SWOT at the macro level for the implementation of exergaming in healthcare

The macro level involves policies and regulations regarding the use of exergaming in healthcare, international collaborations and governmental decisions regarding data storage, use and sharing.

*Strengths:* The participants emphasized the importance of ongoing collaboration between people who are involved in developing regulations, healthcare professionals, companies and technicians. Creating teams consisting of knowledgeable people with diverse backgrounds was the aim of this collaboration. The involvement of technicians in the development of regulations within healthcare was stressed in the following example provided:

Maybe what would help is to have specialists about like technology involved in the government committees to really advise them as well. Sometimes I can imagine that there’s people there who do not have any understanding or knowledge about technology. Therefore, as we are saying like in healthcare, we should work in multidisciplinary teams (...)That is also what we need in the government approach, that would make it be easier. (P1 – a researcher).

The participants characterized the people involved in developing regulations as “free thinkers” and in favour of using innovations in healthcare, and they highlighted that regulations overall support the use of exergames in healthcare. Participants emphasized that any new exergame must comply with healthcare regulations to ensure it is safe for patients and used appropriately in practice.

*Weaknesses:* A weakness indicated at this level was the definition of intended use when developing exergames, during the preparation phase. The participants referred to the challenge that the exergaming ought to be certified before it can be used in healthcare.

One regulatory framework which is intended use, I don’t know if you’re familiar with that. The medical technical products [medical devices] have to be certified. Just for one purpose, and that’s called intended use. (P14 – a person involved in developing regulations within healthcare).

Another weakness was the lack of involvement of patient’s organizations when developing regulations within healthcare. The participants also stressed that allocating responsibilities among different authorities involved in developing healthcare regulations is considered challenging because of the fragmented system.

The main challenge is whether tasks should be handled locally or collaboratively. While doing things independently is faster and easier, it creates problems when data needs to be shared, since others often develop their own separate solutions. This raises a broader national question: should everyone work in the same way, and who should take responsibility – the e-health agency, technology developers, municipalities and regions, the digital development authority, or all stakeholders together? (P17 – a person involved in developing regulations within healthcare).

*Opportunities:* An opportunity mentioned was the existence of governmental funding programs, which support the development of exergames and other digital technologies, in the preparation phase. The participants expressed their satisfaction with the existing healthcare regulations and mentioned that they encouraged the use of exergames. Establishing international collaborations, conducting multicentred studies and creating possibilities for scaling were seen as opportunities that might increase the possibility of receiving governmental funding.

You know, we applied for the big money and then we emphasized, because that is like funding for… innovation. So, then you have to like, you know, take the game. The idea of a game, how can we use games just in general in healthcare? So not only for this, game, but for it has the possibilities of transferring the idea of game for small children into game for bigger children or for other health conditions. (P2 – a nurse and researcher).

*Threats:* A highlighted threat was the challenges in data storage, sharing and ownership. The participants characterized the existing regulations on data storage and sharing as unclear, and P16, a healthcare professional and a researcher, referred to them as “black boxes”. Despite the existing regulations on the use of exergaming in healthcare, the implementation of these regulations was described as challenging by the participants. Participants expressed doubts regarding the implementation of these policies:

But yeah, our experience that it’s not enough to have it in the policy, sometimes it is just in the policy and then maybe there is a lack of something actually happening, but at least, yeah, that’s the beginning. (P22 – a patient representative).

The digitalization of healthcare systems can be time-consuming, and participants mentioned that this process requires continuous governmental investment, which is impossible to ensure in the long run. An overview of the findings is provided in the following table (Table 2).

**Table 2:** Strengths, weaknesses, opportunities and threats of the implementation of exergaming (in the preparation, execution and continuation phases) in healthcare for individuals with chronic conditions at the micro, meso and macro levels

	Strengths	Weaknesses	Opportunities	Threats
Micro level	• Assessing a patient’s needs (P) • Stakeholders’ involvement in development, testing (P) and evaluation of exergaming (E and C) • Explaining exergaming to patients and healthcare professionals (E and C) • Motivating, interesting and simple exergaming (E and C) • Following up with patients in regard to use of exergaming (E and C) • Patients’ independence and willingness in using of exergaming (E and C) • Exergaming adapted into daily life (C)	• Patient’s lack of understanding of exergaming (E and C) • Healthcare professionals lacking confidence with using exergaming (E and C) • Doubts regarding the safety and quality of treatment when using exergaming (E and C)	• Involvement of healthcare professionals (E and C) • Family members’ support (E and C) • Presence of a large pool of exergaming for covering different needs (E and C) • Access to healthcare by exergaming (C)	• Difficulty with involving patients (P) • Exergaming not covering a patient’s needs (E and C) • Healthcare professionals’ perceptions of losing control over exergaming (E and C) • Severe health difficulties of patients (E and C) • Resistance to change (E and C)
Meso level	• Assessing needs within healthcare (P) • Predicting costs (P) • Collaboration between technicians and healthcare professionals (P, E and C) • Sustained communication between collaborators (E and C) • Developing a time plan (E and C)	• Designing exergames being expensive (P) • Technicians, companies and healthcare professionals’ lack of understanding (P, E and C) • The fact that making a profit takes time (E and C) • Exergaming being expensive to sustain (C)	• Existing evidence on exergaming (P) • Use of existing exergaming (P) • Clinics using exergaming in daily practice (E and C) • Supportive leadership (E and C)	• Competition to get research grants (P) • Competition by existing exergaming (P) • Health workforce challenges: staff rotation or turnover and overburdened health workforce (P, E and C) • Not enough time to learn the exergaming (P, E and C) • Not enough evidence about exergaming (P, E and C) • Too many people involved (E and C)
Macro level	• Collaboration between policy-makers, companies, patient’s organizations and technicians (P, E and C) • Following regulations (P, E and C) • Policy-makers support for the use of exergaming (P, E and C)	• Defining of intended use (P) • Lack of collaboration between government and patient’s organizations (P, E and C) • Allocation of responsibilities between different authorities (E and C)	• Governmental funding programs (P) • International collaborations (E and C) • Regulations supporting the use of exergaming (E and C)	• Data storage, sharing and ownership (P, E and C) • Poor compliance with regulations on exergaming in healthcare (C) • Non-digitalized healthcare system (C)

P, E and C stated the phases of the theory of adaptive implementation, and thus the preparation (initiation), execution (actual implementation) and continuation phases (stabilization) [25]. Micro level (direct interactions between patients and professionals), meso level (institutional frameworks), macro level (laws and regulations). Strengths and weaknesses (internal), opportunities and threats (external). P, preparation; E, execution; C, continuation

## Discussion

The implementation of exergaming in healthcare depends upon a multitude of strengths, weaknesses, opportunities or threats at the micro, meso and macro levels and occurs in different phases of implementation (preparation, execution and continuation). An important finding in this study was the importance of the involvement of stakeholders in every phase of implementation. Effective collaboration between people with technical, healthcare and research backgrounds was also considered important when developing, implementing and stabilizing the use of exergames in healthcare. The importance of supportive leadership, high digital literacy of stakeholders and access to technology within healthcare settings were also characterized as strengths in every phase. The lack of scientific evidence regarding the use of existing exergames has created doubts related to patient’s safety. Receiving a governmental grant was considered more likely during the preparation phase, when exergames are being developed, but more challenging to secure during the execution and continuation phases. Despite the regulations supporting the use of exergames and other digital solutions in healthcare, their implementation is often inconsistent in practice. The main findings were discussed in relation to the internal (strengths and weaknesses) and external factors (opportunities and threats) of the SWOT analysis.

### Internal factors (strengths and weaknesses)

The involvement of stakeholders when developing, testing and evaluating exergames was considered highly important by the participants of our study. Their constant involvement provides the possibility to develop exergames tailored to their needs [34]. Continuous collaboration with stakeholders, such as healthcare professionals, patients, game developers and researchers, keeps designing efforts on track and contributes to shaping game design, therapeutic goals and user engagement strategies, according to the results of a previous study [35]. Continuous engagement also facilitates iterative improvements and helps bridge the gap between technical feasibility and practical application in real-world settings [19]. In our study, stakeholder’s involvement in every phase of the process provided relevant feedback, which led to the development of improved exergames and reduced the overall cost of the project. Patient representatives who participated in our study thought exergames to be fun and engaging especially when they were simple and tailored to their needs.

However, the participants of our study mentioned that it can be challenging to involve patients because chronic conditions and care needs are strongly influenced by contextual factors and social aspects. For example, type 2 diabetes, heart failure, cognitive impairment and general frailty are more than twice as common in the poorest and least well-educated quintiles of society as in the richest and best educated, according to the WHO [36]. This population may also have less access to research and technology, potentially leading to lower digital literacy [37].

Effective collaboration between technicians who develop exergames, healthcare professionals and people involved in regulations within healthcare was significant in all the different phases of implementation, as described in a literature review in which collaboration and fruitful communication between people with diverse backgrounds were the most important factors influencing the effective implementation of technology fall prevention [38]. However, the participants in our study highlighted challenges in communication between different groups of professionals, primarily due to differences in academic culture.

The participants in our study referred to several weaknesses that might hinder exergaming’s widespread clinical adoption, potentially leading to patient’s limited adherence and engagement. Early exergaming research has faced several limitations, including the reliance on commercial platforms such as Wii Fit or Kinect that were not tailored to the patients with cardiovascular or neurological conditions, which resulted in inadequate training intensities or irrelevant content [39, 40]. Many of these systems also lacked essential educational components that are central to multidisciplinary cardiac rehabilitation, such as self-management strategies, medication adherence and lifestyle counselling [40]. High-end VR systems and motion-sensing devices are costly and often inaccessible within healthcare, while older adults frequently face digital literacy challenges [41]. Participants in our study also referred to the challenge of sustaining the implementation of a digital solution, due to limited financial support. Long-term engagement has proven difficult as well, with novelty effects wearing off over time and patients reporting boredom, frustration or discouragement, compounded by limited structured follow-up and varying levels of digital literacy [42, 43]. In addition to these issues, safety and monitoring remain major concerns [44].

In the context of exergaming the term “safety” may include risks, such as physical injury due to unsupervised use, overexertion in patients with cardiovascular disease and inaccurate or unreliable feedback. In our study, participants also expressed doubts regarding the safety and quality of exergames as a treatment modality. Previous research suggests that developing exergames tailored into patient’s needs can serve as a safety measure as much as an effectiveness strategy. For example, Blasco-Peris et al. (2025) introduced the HEFMOB system for early cardiac rehabilitation, which individualizes exercise intensity through clinician-set heart rate targets and adaptive resistance, ensuring patients train within safe and effective limits [45]. Likewise, Emre et al. (2025) described a robotic stepper with integrated exergames that allows progression tailored to patient abilities, supporting both safety and functional recovery [46]. These findings demonstrate the importance of developing purpose-built, personalized systems with integrated monitoring and adaptive control mechanisms so that patients avoid both undertraining and harmful overexertion.

### External factors (opportunities and threats)

The significance of supportive leadership within the healthcare setting was emphasized mostly in the execution and continuation phase. This finding is in line with the results of a previous qualitative study that revealed that unsupportive leadership was a main reason for not implementing the nurse-on-call model in primary care [47]. Leadership is complex with respect to social interactions, and it involves essential practices, including initiating, power-sharing, training, supporting, establishing trust, communicating, networking, orchestration and implementation [48]. Leadership and clear purpose are central aspects when introducing and implementing digital solutions in healthcare, such as exergaming [49, 50]. Both management and staff need to have a common understanding of the reasons why the organization needs to change and what benefits might bring to patients.

The participants of our study indicated that existing regulations support the overall use of exergaming and other digital technologies in healthcare. Leaders at the international level have pledged to increase international collaboration to prevent and manage chronic conditions, with a focus on sharing best practices in health promotion, policy, regulation, workforce training and healthcare infrastructure [51]. However, there is a need for developing and following clear frameworks regarding secure data management [52]. Handling patient data in exergames raises potential concerns about privacy.

The participants of our study highlighted that the implementation of these regulations in practice is scarce, leading to challenges in implementing exergames in the continuation phase. This challenge is even more pronounced internationally because regulatory frameworks differ substantially across regions. For example, under Europe’s former Medical Device Directive (MDD), many exergames or digital health apps could be certified as medical devices with relatively limited evidence [18], whereas the US Food and Drug Administration (FDA) generally applies stricter requirements, demanding more robust proof of safety and effectiveness before granting market approval [53].

### Implementation strategies and practical implications

Building on these findings, several strategies could facilitate the effective implementation of exergaming in healthcare. Hospitals and clinics might start with pilot programs or co-design processes involving patients, healthcare professionals and other stakeholders to ensure that exergames are tailored and integrated into clinical workflows. Leadership support is critical, as discussed by the study participants and appointing “digital champions” or dedicated task forces can assist the adoption and maintain momentum. Clear ownership of exergaming initiatives can also help implementation, for instance, by assigning a coordinator to oversee responsibilities, which will reduce fragmentation. Providing healthcare professionals with structured training sessions and protected time to engage with the technology is another key strategy, addressing the commonly reported barrier of insufficient time or confidence to use new tools.

The main contribution of this study lies in providing knowledge to healthcare professionals, game developers, patients, researchers and policy-makers regarding the different factors that influence the implementation of exergaming in healthcare. To our knowledge, this is the only study that attempts to map the entire process of implementing exergames in a comprehensive manner. This study can serve as a valuable resource in guiding the development and implementation of exergaming interventions, increasing the likelihood of their success. These qualitative insights could be used as a foundation for the co-creation of implementation guidelines or testing specific strategies in pilot implementations. Future research should focus on implementation trials or cost–effectiveness studies to successfully address these gaps.

### Strengths and limitations of the study

There are several strengths and limitations of this study. The data were collected from countries that varied with respect to their geographical nature and administrative, political, social, cultural, environmental, economic and healthcare structure. Implementation depends upon contextual factors, and thus, this should be taken into consideration when interpreting the results. However, involving participants from different countries, with distinct academic backgrounds and various experiences with exergaming and other digital technologies aimed at providing a broader understanding of the phenomenon, increases the possibility of transferability. It should be noted that transferability is always shaped by a specific population, context and time of the study [54]. Since this study focused on exergaming among individuals with chronic conditions, this context should be carefully considered when interpreting the results to avoid overgeneralization.

Conducting data collection digitally via Zoom interviews has many strengths, such as convenience and ease of use, an enhanced personal interface to discuss personal topics and accessibility (that is, phone, tablet and computer), and this method saves time, with no travel requirements [55, 56]. In this study, the online conference system made it possible to interview participants globally, and the process ran smoothly with no significant distractions or internet connectivity issues reported.

The confirmability of the study was strengthened by transparently reporting the process of data collection and analysis. Using participant’s direct quotes ensured that their actual insights and experiences were reflected. Since the interview guide was based on the SWOT framework, all the data collected aligned with it. SWOT analysis provides a clear structure and facilitates qualitative descriptive and cross-sectional analysis [22]. The purposeful and snowball sampling strategy strengthened the trustworthiness of the findings since they led to richer data which provided a deeper understanding of the use of exergaming. Credibility was facilitated via investigator triangulation since different researchers were involved in the process of data analysis [57]. Researchers constantly reflected upon their own biases and preconceptions that could potentially influence their decisions and actions. Member checking that involved asking a participant to verify the interview transcription was also performed to increase study credibility and confirmability.

## Conclusions

In conclusion, being aware of the factors that can potentially act as strengths, weaknesses, opportunities or threats is meaningful when developing and implementing exergaming in healthcare. This study highlights the complex and multifaceted nature of implementing exergames in healthcare, emphasizing the need for coordinated efforts across the technical, clinical and research domains. The findings offer valuable insights into how stakeholder and patient involvement, supportive leadership and digital readiness can enhance implementation success while also identifying key barriers such as inconsistent policy application and limited evidence on patient safety. These insights can inform future strategies for more effective and sustainable integration of exergaming in healthcare settings.

## Supplementary Information

Supplementary material 1.

## Data Availability

By contacting the corresponding author, procedures for sharing data, analytic methods and study materials for reproducing the results or replicating the procedure can be arranged following Swedish legislation.

## Abbreviations

JA-CHRODIS: Joint Action on Chronic Diseases and Promoting Healthy Ageing across the Life Cycle
SWOT: Strengths, weaknesses, opportunities and threats
MDR: Medical devices regulation
MDD: Medical device directive
FDA: Food and Drug Administration
