# Supplementary material for: Implementing exergames into healthcare for chronic conditions – insights from stakeholders: a qualitative study
Source: Health Res Policy Syst. 2025 Oct 30;23:144. doi: 10.1186/s12961-025-01416-7 (PMC12577426; doi:10.1186/s12961-025-01416-7)
Supplement: Supplementary file 1 — Supplementary material 1. [file 12961_2025_1416_MOESM1_ESM.docx]

**Appendix- Definition of exergaming and interview guide**

***Exergame (definition)***

Exergaming can be defined as playing a videogame by using full body movement to control on-screen action, and it requires the player to expend a significantly greater amount of energy than resting levels do. Thus, this type of gaming combines physical exercise and the use of virtual reality and technology. Video game consoles, virtual reality systems, or mobile applications are used to support physical activity and provide a more interactive experience (e.g. you might have heard of Nintendo Wii or cybercycling).

1. ***Internal factors (strengths-weaknesses):***

- Could you describe your experience of developing/designing/using exergaming?
- Could you tell me about the use of exergaming in your organization?
- Could you tell me about competencies (knowledge or skills about the new technology) so that exergames are used?
- Could you elaborate on what you think could be the advantages of using exergaming in healthcare?
- What do you think could be the challenges of using exergaming in healthcare?
- How much support (and, what kind of support, from whom) do you/your organization/company need to use exergaming? How much time is needed? Are there enough resources? (resources might be: time, technical support, cost, access to evidence/quality of evidence, management support, schedule issues, and education).
- Do you think that the potential benefits of exergaming outweigh the required resources?
- Do you think that your organization meets, has arranged, or has introduced appropriate conditions for exergaming? *What should be added or removed from the environment or organization so that exergaming can be used? Which factors discourage and encourage users in exergaming in their organization?*

Now we discussed the factors influencing the use of exergaming in healthcare within the organization and I would like us to discuss the factors that might influence the outside of the organization.

***2. External factors(opportunities-threats):***

- How could the use of exergaming in healthcare be achieved?
- Do you think that regulations and policies could affect the use of exergaming in healthcare? And if yes, in what way?

Probing: Do you think that the use of exergaming is supported by the policies? If yes, in what way? If not, please provide examples and mention what changes are needed.

- How could the use of exergaming in healthcare be maintained? What is needed? Please provide examples.

Thank you very much for your participation!
